# Concentration-Dependent Evolution of the Belousov–Zhabotinsky Reaction as Determined by X‑ray Absorption and UV–Vis Spectroscopies

**DOI:** 10.1021/acs.jpcb.5c04472

**Published:** 2025-10-03

**Authors:** Francesco Tavani, Giorgio Capocasa, Marika Di Berto Mancini, Federico Frateloreto, Daniele Del Giudice, Osvaldo Lanzalunga, Stefano Di Stefano, Paola D’Angelo

**Affiliations:** Dipartimento di Chimica, 9311 Università degli Studi di Roma La Sapienza, P.le A. Moro 5, I-00185 Rome, Italy

## Abstract

Although the Belousov–Zhabotinsky (BZ) chemical reaction has been the object of intense research efforts for almost a century, many aspects of the BZ complex oscillatory behavior still remain to be clarified, also due to difficulties in experimentally monitoring the speciation of the main brominated compounds during the reaction cycles. Herein, we describe an integrated approach based on Br K-edge X-ray absorption and ultraviolet–visible (UV–vis) spectroscopies to identify the onset and evolution of concentration-dependent collective bromine oscillations in the classical BZ reaction. Principal component analysis, multivariate curve resolution, and theoretical X-ray spectroscopy simulations were combined to identify the number, nature, and concentration time evolution of the key reaction brominated species during the chaotic and periodic BZ regimes. Our integrated approach enabled real-time monitoring of how variations in metal catalyst concentration influence both the metal center and key brominated BZ species throughout the different stages of the complex reaction pathway. The multidisciplinary experimental and theoretical approach, sensitive to both the brominated and metal portions of the BZ system, overcomes the challenges in detecting the spectroscopically silent BZ reaction species and may be applied to rationalize a wide range of BZ and non-BZ oscillatory reactions.

## Graphical abstract

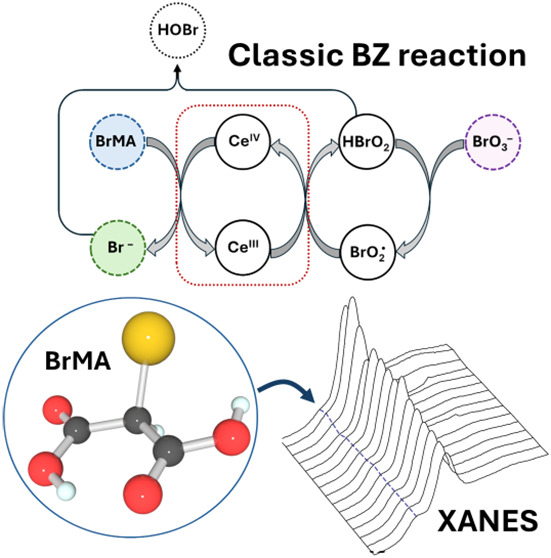

## Introduction

The Belousov–Zhabotinsky (BZ) reaction is an intriguing chemical process famous for its nonlinear oscillatory behavior kept far from the thermodynamic equilibrium.^1–4^ Oscillations in the BZ reaction are sustained through the oxidation of organic compounds by a mixture of a metal catalyst, malonic and sulfuric acids, and bromate,^1^ resulting in the following overall equation^1,5^

1 $$3{\mathrm{CH}}_{2}{\left(\mathrm{COOH}\right)}_{2}+4{\mathrm{BrO}}_{3}^{-}\to 4{\mathrm{Br}}^{-}+9{\mathrm{CO}}_{2}+6H_{2}O$$

While the classical BZ reaction involves the cerium-catalyzed bromate oxidation of citric acid,^1^ other BZ oscillating systems based on different redox-active metals (such as iron,^6,7^ manganese,^8,9^ copper,^10^ and ruthenium^11^) and dicarboxylic acids have been reported. It has also been shown that BZ processes may be controlled through the variation of parameters such as reaction temperature, proportion of reactants,^12^ illumination, or stirring rate.^13^ Notably, the dynamics of an unstirred BZ system become more complex as diffusion and convection effects significantly contribute to the reaction kinetics,^14,15^ often resulting in chaotic behavior^16^ with chaotic transient phases being observed in between periodic phases during cerium- and iron-catalyzed BZ reactions.^17–19^ Although intense research efforts have been devoted to unravelling the BZ mechanism^1,3,4,20–35^ and harnessing BZ oscillatory systems for applications in chemical computing,^36^ material engineering,^37,38^ and catalysis,^39^ numerous aspects of the BZ complex oscillatory behavior have so far eluded conventional methods of detection and still remain to be clarified. The key steps of the BZ mechanism proposed by Field, Köros, and Noyes (FKN)^21^ about half a century ago involve the consumption of bromate, the autocatalytic formation of the BrO_2_^•^ radical, resulting in the metal catalyst oxidation, and the decarboxylative oxidation of the organic substrate, which leads to the reduction of the metal catalyst and new bromide production (see Figure 1a).^7^ The FKN model (Table S1) is derived from chemical kinetic equations, and its periodic solutions aim to reproduce the BZ nonchaotic oscillation regime,^5^ with more complex theoretical models replicating chaotic motion^40^ or the BZ reaction spatial patterns.^41^ Nevertheless, a major obstacle in the way of unravelling the intricate mechanisms of stirred and unstirred BZ oscillators has been the experimental challenge of tracking the evolution of the main BZ brominated species, since bromine compounds are largely silent to conventional spectroscopic techniques.^42,43^

**Figure 1:**
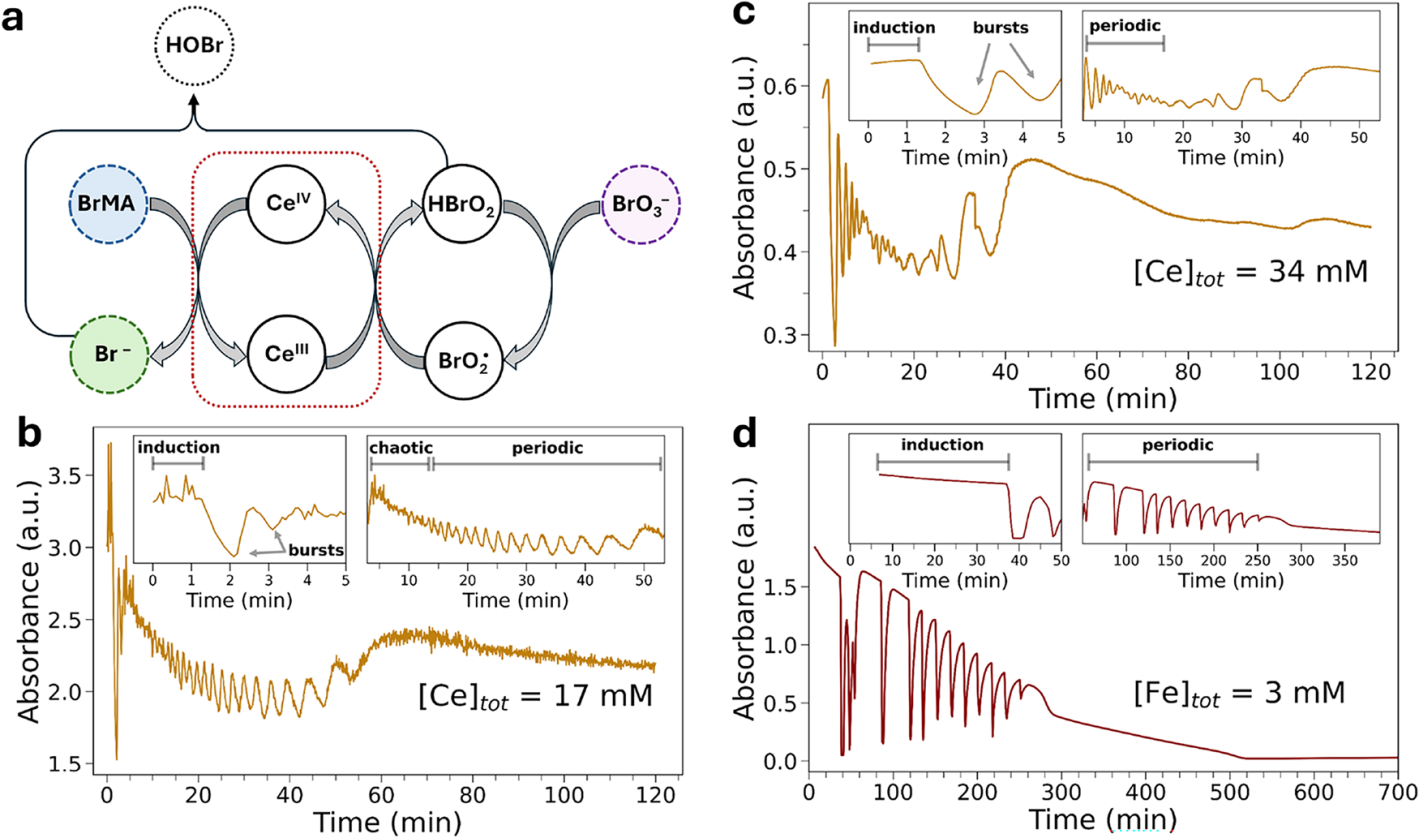
(a) Schematic overview of the FKN key processes that take place during the cerium-ion-catalyzed BZ reaction. (b, c) UV–vis time monitoring at λ = 420 nm of the BZ reaction catalyzed by Ce 17 mM (b) and Ce 34 mM (c). (d) UV–vis time monitoring at λ = 509 nm of the BZ reaction catalyzed by Fe 3 mM. Magnifications of the plots are provided in the insets.

X-ray absorption spectroscopy (XAS) stands out as an advanced probe to overcome experimental difficulties in detecting the evolution of the brominated portion of BZ systems. XAS is an established method for studying the local electronic and structural properties of chemical systems in the liquid state,^44^ such as chemical reactions occurring on the minute^45^ to millisecond time scales.^46^ By employing time-resolved XAS, we have recently shown that collective large-amplitude oscillations of the brominated species are present and absent in BZ reactions carried out in the presence of relatively high^47^ ([Ce_tot_] ∼ 34 mM) and low^48^ ([Fe_tot_] ∼ 3 mM) metal catalyst concentrations.

These previous insights motivated our efforts to answer the following questions on the behavior of BZ systems: (i) What is the relationship between metal concentration and collective bromine oscillations? (ii) How does this relationship influence patterns in the concentration time evolution of the key brominated species during both the reaction chaotic and periodic regimes? In order to address these scientific questions, we resort herein to time-resolved Br K-edge XAS and comprehensively characterize the bromine chemistry of the classical BZ reaction carried out in an unstirred reactor and at a cerium ion concentration of 17 mM, conditions that we found to be suitable to pinpoint and interpret the onset of collective bromine oscillations. We complement the XAS measurements with UV–vis spectroscopy to contextually follow the Ce^4+^ to Ce^3+^ oscillatory transformation, while utilizing principal component analysis (PCA), multivariate curve resolution (MCR), and *ab initio* theoretical XAS simulations to determine the number, nature, concentration time evolution, and structure of the BZ key brominated components.

## Experimental Section

### BZ Reaction Conditions

Three systems were studied: (i) Ce-based BZ with 17 mM Ce­(NH_4_)_4_(SO_4_)_4_ in 0.48 M H_2_SO_4_, 80 mM NaBrO_3_, 8 mM KBr, and 50 mM malonic acid (MA); (ii) Ce-based BZ with 34 mM Ce­(NH_4_)_4_(SO_4_)_4_ in 1.7 M H_2_SO_4_, 70.2 mM NaBrO_3_, 5.6 mM KBr, and 70 mM MA; and (iii) Fe-based BZ with 3 mM ferroin in 0.48 M H_2_SO_4_, 80 mM NaBrO_3_, 8 mM KBr, and 50 mM allylmalonic acid. All reactions were performed at 25 °C in initially homogenized unstirred aqueous solutions. UV–vis measurements were carried out in a custom quartz cell (52 mm × 9.5 mm × 1 mm), while XAS experiments used an aluminum alloy cell of identical dimensions sealed with Kapton windows.

### X-ray Absorption Measurements

Br K-edge XAS spectra were collected at room temperature in the transmission mode at the XAFS beamline of the Elettra Synchrotron (Trieste, Italy), with the storage ring operating at 2 GeV and a beam current of 130–300 mA. Samples were loaded in an aluminum alloy cell sealed with 0.5 mm Kapton windows, and time-resolved XANES was acquired in the fast-scan mode. Data were normalized using Athena,^49^ and plots were generated with custom Python scripts.

## Results and Discussion

We began our investigation by following a cerium-ion-catalyzed BZ reaction and employing independent UV–vis and Br K-edge XAS measurements. The BZ reaction was carried out in an unstirred reactor cell (see Figure S1), where malonic acid (MA, 70 mM), H_2_SO_4_ (1.75 M), NaBrO_3_ (70.2 mM), KBr (5.6 mM), and Ce­(NH_4_)_4_(SO_4_)_4_ (17 mM) were mixed in aqueous solution at room temperature (RT). In particular, the reactants were mixed in the UV–vis cell shown in Figure S1 with height, inner width, and inner depth dimensions equal to 52, 9.5, and 1 mm, respectively. The UV–vis kinetic trace recorded at 420 nm under these experimental conditions evidenced that periodic oscillations of the concentration of Ce^4+^ occur during the BZ reaction, as shown in Figure 1b.^47^ Based on the UV–vis reaction monitoring, one may observe that after an induction time of 1.3 min, two consecutive oscillations with relatively larger amplitudes occur at *t* = 2 min and *t* = 3 min from the start of the reaction (see the inset of Figure 1b) and rapidly deplete after *t* = 4 min. These more pronounced oscillations may be attributed to the initial “bursts” in the Ce^4+/3+^ concentration.^47^ This initial phase is then followed by a chaotic phase (see the inset of Figure 1b), where the UV–vis oscillations are not well-defined and extend up to ∼10 min. Subsequently, the BZ reaction enters the periodic phase and the UV–vis trace at 420 nm exhibits well-defined oscillations whose time periods increase as a function of time up to *t* ∼ 60 min,^47^ after which the oscillatory behavior is depleted as the reaction comes to a halt. In order to evidence how the metal catalyst concentration affects the BZ behavior, we compared these results to previously collected^47,48^ UV–vis kinetic measurements of BZ reactions carried out in unstirred reactors and in the presence of cerium and iron ion concentrations of 34 and 3 mM, respectively (see Figure 1c,d, respectively). Note that it was not possible to use cerium at a very low concentration due to the lower molar extinction coefficient, which hampered the UV–vis detection of the reaction. Looking at Figure 1, one may observe that in the cerium 34 mM BZ reaction after an induction time of ∼1.3 min, two large-amplitude UV–vis oscillations occur at *t* < 5 min, after which the reaction enters the periodic regime, with nonperiodic behavior being observed for *t* > 38 min. Importantly, the oscillatory behavior of the cerium 34 mM reaction comes to a halt earlier than that of the cerium 17 mM reaction. Conversely, the iron 3 mM BZ reaction (see Figure 1d) starts after a more prolonged induction time of about 10 min, after which a periodic phase occurs, which is characterized by regular oscillations whose periods are longer than those of the UV–vis oscillations observed in the cerium-catalyzed BZ processes. One may also observe that the oscillations of the iron 3 mM reaction come to a halt after ∼300 min from the start of the reaction,^48^ which is considerably longer than that observed for the Ce-based BZ transformations. These findings highlight how an increase in metal catalyst concentration affects the BZ behavior by (i) accelerating the reaction kinetics and (ii) shortening the duration of the reaction stage when periodic oscillations in the metal concentration occur.

We then employed Br K-edge XAS, an intrinsically element-selective technique,^50^ to reveal how the structural and electronic properties of the key brominated species evolve during the BZ process. To this end, we collected time-resolved Br K-edge XAS spectra of the BZ reaction in the transmission mode at the Elettra Synchrotron (Trieste, Italy) on the XAFS beamline. In particular, we mixed Ce­(NH_4_)_4_(SO_4_)_4_ (17 mM), MA (70 mM), H_2_SO_4_ (1.75 M), NaBrO_3_ (70.2 mM), and KBr (5.6 mM) in aqueous solution at room temperature within a cell having the same dimensions as those of the cell used during the previous UV–vis experiment. Figure 2a shows a 3D depiction of the time-resolved Br K-edge XAS spectra collected during the first 7 min of the BZ reaction and displayed in the X-ray absorption near edge structure (XANES) energy region. Notably, the time evolution of the intensity of the white line transition at 13477.7 eV (Figure 2a, dashed red line) displays an oscillatory behavior during the initial reaction stage, exhibiting a relative increase, decrease, and new increase at *t*_1_ = 2.3 min, *t*_2_ = 2.6 min, and *t*_3_ = 3.0 min, respectively (see highlighted time points in Figure 2a). We note that these intensity variations extend to all of the XANES spectra and may be attributed to collective oscillations in the concentration of the main brominated species interchanging during the BZ reactive pathways, which are linked to the concentration “bursts” of Ce^4+/3+^ highlighted by UV–vis.^47^ Indeed, we have recently highlighted similar, albeit more intense, oscillations in the XAS spectra while monitoring the classical BZ reaction carried out in the presence of a 34 mM cerium.^47^ A set of time-resolved XANES spectra measured during the BZ reaction investigated here is shown in Figure 2b, where selected XAS spectra collected at *t* = 3.0 min, *t* = 56.3 min, and *t* = 143.8 min are presented in dark red, blue, and green, respectively. We point out that the first and last experimental XANES spectra (Figure 2b, dark red and green) bear a strong resemblance to those of 0.1 M aqueous solutions of NaBrO_3_ and of KBr, respectively (the XAS spectra are compared in Figure S2). This observation is in agreement with the fact that BrO_3_^–^ and Br^–^ are expected to be the prevalent brominated species in the reaction mixture at short and prolonged reaction times, respectively. One may note that during the first stage of the reaction (*t* < 57 min), a pre-edge peak at 1372.5 eV appears. As the reaction proceeds further (*t* > 57 min), this feature is gradually depleted, supporting the view that a brominated intermediate species is first formed and then progressively consumed during the BZ process. Furthermore, as the intermediate brominated species is produced and consumed, two distinct isosbestic points are detected in the XANES spectra at 13472.8 and 13473.5 eV, respectively, further suggesting that at least three main brominated compounds prevalently contribute to the experimental XAS data.

**Figure 2:**
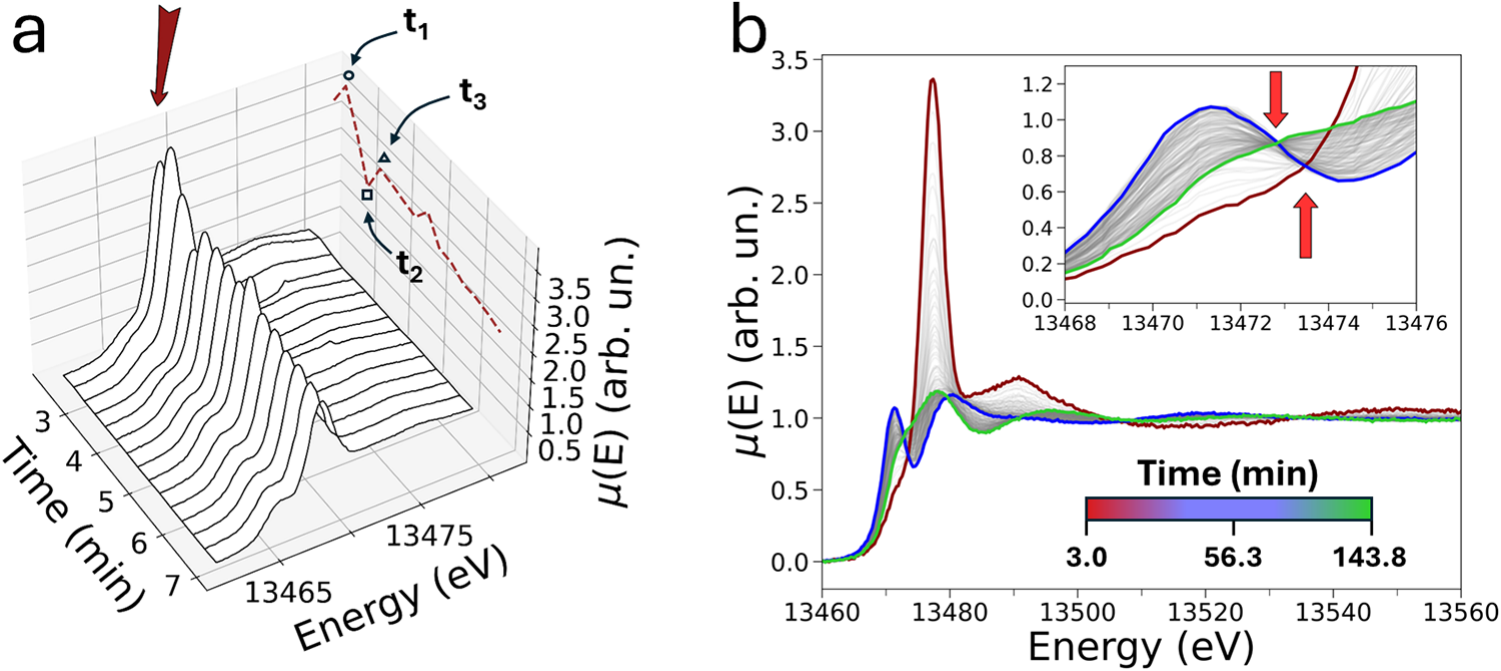
Time evolution of the Br K-edge XANES spectra recorded during the BZ reaction catalyzed by Ce 17 mM. (a) 3D representation of the Br K-edge XANES spectra. A dark red arrow and dotted line highlight the transition located at 13477.7 eV and the time evolution of the same feature, respectively. (b) 2D depiction of the time-resolved Br K-edge XANES spectra. An enlargement of the pre-edge region is shown in the inset, with arrows indicating two isosbestic points at 13472.8 and 13473.5 eV.

We next performed a PCA of the time-resolved Br K-edge XAS data set to obtain quantitative information on the number of distinct components contributing to the experimental spectra.^46^ PCA is an unsupervised machine learning technique that has been fruitfully applied to identify the smallest number of independent components, often referred to as principal components (PCs), whose linear combination best explains the variance in XAS time-resolved measurements.^46,51–53^ Following the Lambert–Beer law, the number of brominated chemical species prevalent during the BZ reaction and contributing to the XAS measurements may be identified as the number of PCs required to explain the variance in the XAS data within statistical error.^46,50^ We began our analysis by subjecting the time-resolved XANES data to a scree plot statistical test and determined the number N of PCs contributing to the XAS data to be 3 (Figure 3a). There is, in fact, a characteristic elbow in the scree plot between *N* = 3 and *N* = 4, indicating that for *N* > 3, the associated PCs negligibly contribute to the time-resolved XAS spectra. We then reconstructed the XANES data set with *N* = 3 and found that the percentage residual error never exceeds 0.02% in the reconstruction of distinct spectra (see Figure 3b) and therefore may be considered negligible. Subsequently, we focused on gaining quantitative knowledge on the identity of the key brominated reaction species and their concentration time evolution as the BZ reaction proceeds. To this end, we subjected the XANES data to an MCR analysis by employing 3 PCs and constraining the first two spectral components to be equal to the XANES spectra of the Br^–^ and BrO_3_^–^ reference aqueous solutions.^47,48^ We report the retrieved XANES spectral and concentration profiles in Figure 3c,d, respectively. As shown in Figure 3c, the XAS spectra of the Br^–^ and BrO_3_^–^ components exhibit marked differences with that of BrO_3_^–^, displaying, for instance, a significantly more intense white line transition compared to that of Br^–^. Importantly, the MCR analysis evidenced that the XAS spectrum of the third reaction component (blue curve, Figure 3c) possesses a pre-edge transition at 13472.5 eV in analogy to the XANES spectrum of a 0.1 M methanol solution of diethyl bromomalonate, which is shown in Figure S3. We have recently highlighted the presence of this feature, which is known to be due to a 1s → 4p dipole-allowed transition,^54–59^ in the XANES spectra of bromomalonic acid (BrMA)^47^ and bromoallylmalonic acid (BrAMA),^48^ which are brominated species that form when the BZ reaction is conducted in the presence of malonic and allylmalonic acid, respectively. This evidence led us to identify the third component as BrMA with good confidence.^47^ To confirm this hypothesis, we performed an *ab initio* theoretical simulation of the XAS spectrum of BrMA starting from a cluster model (shown in Figure 4) optimized by means of density functional theory (DFT) with the ORCA program.^60^ The theoretical XAS spectrum of the BrMA species was then simulated starting from the DFT-optimized molecular geometry by means of the FDMNES code,^61,62^ making use of the approximated muffin-tin potential and including quadrupolar transitions in the XAS calculations. A cutoff radius of 8.2 Å was employed to include all of the BrMA atoms in the XAS simulations. The XANES spectra of the MCR-extracted BrMA species and the theoretical XANES curve are compared in Figure 4 and are in very good agreement. In particular, the energies and relative intensities of both the pre-edge and white line experimental transitions are excellently reproduced by the calculations, thereby further supporting our assignment of the third brominated reaction component to be BrMA. It should be noted that the presence of additional species such as Br_2_, HBrO_2_, HOBr, or bromoacetic acid cannot be excluded; however, their concentrations are likely to be below the detection limit of the XAS technique. This observation is consistent with our previous findings.^47,48^

**Figure 3:**
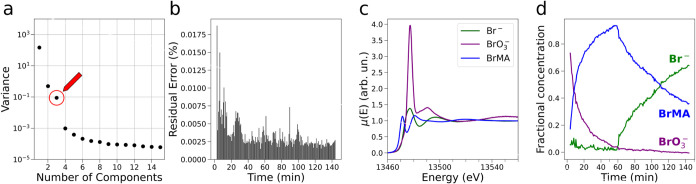
PCA and MCR analyses of the Br K-edge XAS spectra recorded during the BZ reaction catalyzed by Ce 17 mM. (a) Scree plot of the XAS data, where a red arrow and circle evidence the singular value associated with *N* = 3. (b) Residual error committed while reconstructing the XAS data with *N* = 3 PCs. (c) MCR-extracted XANES spectra associated with the XAS spectra of Br^–^ (green), BrO_3_^–^ (purple), and BrMA (blue). (d) MCR-extracted concentration profiles of the three XAS spectral components.

**Figure 4:**
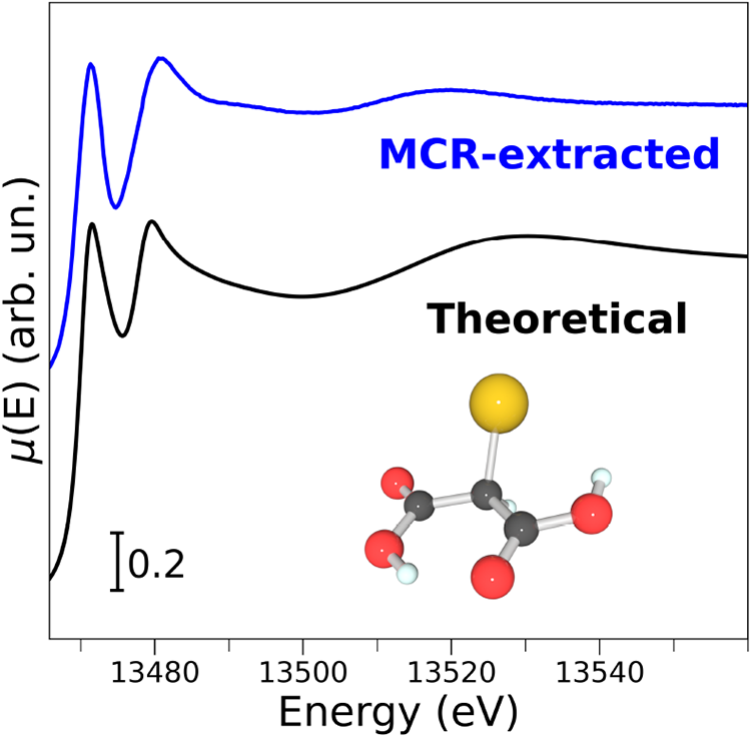
Br K-edge experimental and theoretical XANES spectra of BrMA. The experimental spectrum derived from the MCR analysis is shown in blue, while the calculated curve is shown in black. The minimum-energy structure of BrMA obtained from DFT calculation is also reported, where the bromine, oxygen, carbon, and hydrogen atoms are shown in yellow, red, black, and azure, respectively.

As shown in Figure 3d, the concentrations of BrO_3_^–^ and of BrMA decrease and increase up to ∼10 min at a faster rate and then at a slower rate up to ∼60 min, when the concentration of BrO_3_^–^ is depleted and that of BrMA reaches a maximum value of ∼94%. Notably, the concentration of Br^–^ exhibits more pronounced oscillations between ca. 6 and 10% up to *t* ∼ 10 min, while the oscillations are less pronounced for 10 < *t* < 60 min. These observations imply that the initial reaction phase, where chaotic oscillations of the Ce^4+/3+^ concentration occur (see Figure 1b), is also characterized by a more rapid consumption of BrO_3_^–^, a faster production of BrMA, and concentration oscillations of Br^–^ with relatively large amplitudes and shorter periods. Interestingly, as the BZ reaction enters the reaction phase characterized by the periodic oscillations of the Ce^4+/3+^ concentration (*t* > 10 min, Figure 1b), the concentration oscillations of Br^–^ become less frequent and the Br^–^ relative concentration values fall below 2–3%, suggesting that the variations of the Br^–^ concentration during the periodic reaction phase are barely within the sensitivity of the XAS technique. Further, one may note that during the periodic reaction phase, the decay of BrO_3_^–^ and the buildup of BrMA occur at a slower rate compared to those of the initial chaotic phase. Subsequently, for *t* > 60 min, we observe a progressive increase and decrease of the Br^–^ and BrMA content, respectively, which may be attributed to the progressive processes that lead to Br^–^ from BrMA.^47^

To further corroborate the validity of our statistical analysis and to shed light on the effect of the cerium concentration on the reaction mechanism, Figure 5 compares the experimental XANES spectra recorded at selected times during BZ reactions carried out in the presence of cerium 17 and 34 mM to their reconstructed XANES spectra obtained by using 3 PCs. In all cases, the agreement between the experimental and reconstructed curves is excellent, confirming that 3 PCs predominantly contribute to the time-resolved XANES measurements. Note that the XANES spectra recorded at *t* = 4.6 min and *t* = 28.0 min during the cerium 17 mM BZ reaction show less intense pre-edge features compared to the XANES spectra measured at the same times during the cerium 34 mM reaction. This indicates that the higher amount of metal catalyst leads to a faster production and buildup of BrMA in the cerium 34 mM process. Coherently, the XANES spectra measured at *t* = 68.0 min and *t* = 85.5 min during the cerium 17 mM reaction still display the characteristic pre-edge transition of BrMA, which is instead hardly detectable in the XANES spectra measured at the same reaction times when cerium 34 mM is present in the mixture. Indeed, the higher amount of cerium accelerates the BZ oscillatory cycles and consumes the BrMA species more rapidly. Conversely, the XANES spectra measured at selected times during a BZ reaction carried out in the presence of iron 3 mM (see Figure S4) display a significantly slower pre-edge intensity increase due to the lower concentration of the metal catalyst, which leads to a slower reaction rate.

**Figure 5:**
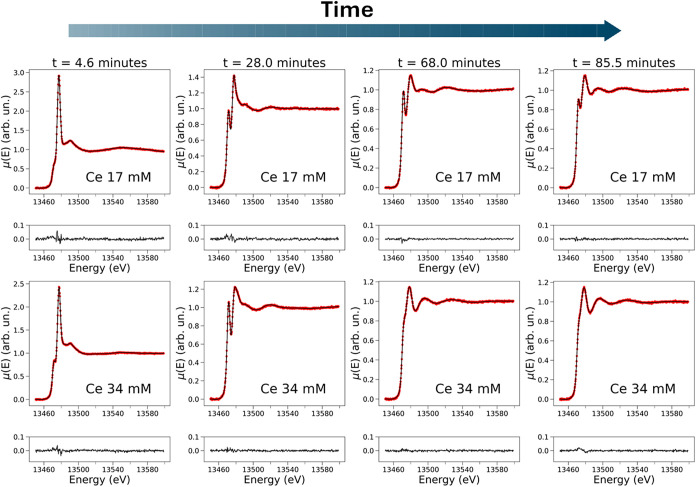
Statistical reconstruction of XANES spectra recorded while carrying out the BZ reaction in the presence of Ce 17 and 34 mM (top and bottom rows, respectively). In each row, selected experimental XANES spectra (dotted red lines) are displayed at given times from the start of the reaction along with their corresponding reconstructed XANES spectra obtained by employing *N* = 3 PCs (solid black lines). The absolute errors between the experimental and reconstructed curves are reported below each XANES spectrum.

The observed behavior of the cerium 17 mM reaction highlights those aspects of BZ chemistry that are influenced by metal catalyst concentration. First, we found that a higher metal concentration (when considering either cerium or iron as a catalyst) increased the reaction kinetics and led to a faster production and consumption of the brominated malonic acid intermediate. Second, we observed that lowering the cerium concentration from 34 mM to 17 mM results in Ce^4+/3+^ concentration oscillatory “bursts”, which were, in turn, coupled to lower-amplitude collective oscillations of the concentrations of the brominated species, as detected by our time-resolved XANES measurements in the initial reaction phase. In addition, reducing the cerium concentration led to the detection of a reaction phase characterized by the ill-defined oscillations of the Ce^4+/3+^ concentration before periodic oscillations were established. A combined PCA and MCR analysis of the XANES data indicated that BrO_3_^–^ and BrMA are consumed and produced, respectively, at a faster rate and that the concentration of Br^–^ exhibits oscillations with higher amplitudes and shorter periods during this chaotic phase compared to the behavior of the same brominated species during the following periodic phase.

## Conclusions

This study demonstrates that metal catalyst concentration-dependent effects on the behavior of the BZ reaction may be profitably monitored using a combined XAS/UV–vis spectroscopic analysis. Our integrated approach allowed us to directly monitor how changes in metal catalyst concentration affect the behavior of both the metal and brominated key BZ species during different stages of the BZ complex reactive pathways. By employing PCA, MCR, and *ab initio* theoretical XAS simulations, we characterized the identity and behavior of BrO_3_^–^, Br^–^, and BrMA during the investigated BZ reaction, a nontrivial result given the fact that the brominated BZ compounds are largely spectroscopically silent. In particular, in our study, we were able to directly pinpoint the onset and evolution of collective bromine oscillations during the cerium 17 mM BZ reaction, demonstrating the elevated potential of our interdisciplinary method to shed light on often elusive BZ chemistry. Further, our approach is a promising methodology to probe and better understand the evolution of BZ systems for practical applications, including their use in chemical computing devices^63^ and in the development of synchronized chemical oscillators.^64^

## Supplementary Material



## Data Availability

The data supporting the findings of this study are available from the authors upon request.

## Notes

Reaction conditions, XAS experimental details, principal component analysis details, and the FKN model (PDF)

## References

[1] Zhabotinskii A. M. (1964). Periodic
Course of the Oxidation of Malonic Acid in a Solution (Studies on
the Kinetics of beolusov’s reaction). Biofizika.

[2] Prigogine, I. Non-Equilibrium Statistical Mechanics; Interscience: New York, 1963.

[3] Sirimungkala A., Försterling H.-D., Dlask V., Field R. J. (1999). Bromination
Reactions Important in the Mechanism of the Belousov Zhabotinsky System. J. Phys. Chem. A.

[4] Field R. J., Körös E., Noyes R. M. (1972). Oscillations in Chemical Systems.
II. Thorough Analysis of Temporal Oscillation in the Bromate-Cerium-Malonic
Acid System. J. Am. Chem. Soc..

[5] Karimov A., Kopets E., Karimov T., Almjasheva O., Arlyapov V., Butusov D. (2023). Empirically developed
model of the
stirring-controlled Belousov-Zhabotinsky reaction. Chaos Solit. Fractals.

[6] Sobel S. G., Hastings H. M., Field R. J. (2006). Oxidation State
of BZ Reaction Mixtures. J. Phys. Chem. A.

[7] Zars E., Glaser R., Downing M., Chicone C. (2018). Measurements and Simulations
of the Acidity Dependence of the Kinetics of the Iron-Catalyzed Belousov-Zhabotinsky
Reaction: Proton-Catalysis in the Electron Transfer Reaction Involving
the [Fe­(phen)_3_]^3+^ Species. J. Phys. Chem. A.

[8] Lin H.-P., Jwo J.-J. (1995). Kinetic Study of the Belousov-Zhabotinskii
Reaction
with Phenylmalonic Acid. J. Phys. Chem. A.

[9] Doona C. J., Kustin K., Orban M., Orban M., Epstein I. R. (1991). Systematic
Design of Chemical Oscillators. 74. Newly Designed Permanganate-Reductant
Chemical Oscillators. J. Am. Chem. Soc..

[10] Hu G., Chen L., Zhang J., Chen P., Wang W., Song J., Qiu L., Song J., Hu L. (2009). Determination
of Alizarin Red S Using a Novel B-Z Oscillation System Catalyzed by
a Tetraazamacrocyclic Complex. Cent. Eur. J.
Chem..

[11] Delgado J., Zhang Y., Xu B., Epstein I. R. (2011). Terpyridine- and
Bipyridine-Based Ruthenium Complexes as Catalysts for the Belousov-Zhabotinsky
Reaction. J. Phys. Chem. A.

[12] Gentili P. L., Horvath V., Vanag V. K., Epstein I. R. (2012). Belousov-Zhabotinsky
“Chemical Neuron” as a Binary and Fuzzy Logic Processor. Int. J. Unconv. Comput..

[13] Kalishyn Y. Y., Rachwalska M., Strizhak P. E. (2010). Stirring Effect on the Belousov-Zhabotinsky
Oscillating Chemical Reactions in a Batch. Experimental and Modelling. Z. Naturforsch. A.

[14] Wodlei F., Hristea M. R., Alberti G. (2022). Periodic Motion in the Chaotic Phase
of an Unstirred Ferroin-Catalyzed Belousov Zhabotinsky Reaction. Front. Chem..

[15] Biosa G., Masia M., Marchettini N., Rustici M. (2005). A Ternary Nonequilibrium
Phase Diagram for a Closed Unstirred Belousov-Zhabotinsky System. Chem. Phys..

[16] Onuma H., Okubo A., Yokokawa M., Endo M., Kurihashi A., Sawahata H. (2011). Rebirth of a Dead Belousov-Zhabotinsky
Oscillator. J. Phys. Chem. A.

[17] Rustici M., Branca M., Caravati C., Marchettini N. (1996). Evidence of
a Chaotic Transient in a Closed Unstirred Cerium Catalyzed Belousov-Zhabotinsky
System. Chem. Phys. Lett..

[18] Rossi F., Budroni M. A., Marchettini N., Cutietta L., Rustici M., Liveri M. L. T. (2009). Chaotic Dynamics
in an Unstirred Ferroin Catalyzed
Belousov-Zhabotinsky Reaction. Chem. Phys. Lett..

[19] Rustici M., Branca M., Brunetti A., Caravati C., Marchettini N. (1998). Inverse Ruelle-Takens-Newhouse
Scenario in a Closed Unstirred Cerium-Catalysed Belousov-Zhabotinsky
System. Chem. Phys. Lett..

[20] Fujieda S., Mogami Y., Moriyasu K., Mori Y. (1999). Nonequilibrium/nonlinear
chemical oscillation in the virtual absence of gravity. Adv. Space Res..

[21] Field R. J., Noyes R. M. (1974). Oscillations in Chemical Systems.
IV. Limit Cycle Behavior
in a Model of a Real Chemical Reaction. J. Chem.
Phys..

[22] Luo Y., Epstein I. R. (1991). Systematic Design
of Chemical Oscillators. 69. A General
Model for pH Oscillators. J. Am. Chem. Soc..

[23] Epstein I. R., Showalter K. (1996). Nonlinear
Chemical Dynamics: Oscillations, Patterns,
and Chaos. J. Phys. Chem. A.

[24] Vanag V. K. (2004). Waves and
Patterns in Reaction-Diffusion Systems. Belousov-Zhabotinsky Reaction
in Water-in-Oil Microemulsions. Phys.-Usp..

[25] Noszticzius Z., Farkas H., Schelly Z. A. (1984). Explodator:
ANew Skeleton Mechanism
for the Halate Driven Chemical Oscillators. J. Chem. Phys..

[26] Ruoff P., Varga M., Körös E. (1988). How Bromate Oscillators Are Controlled. Acc. Chem. Res..

[27] Cassani A., Monteverde A., Piumetti M. (2021). Belousov-Zhabotinsky Type Reactions:
the Non-linear Behavior of Chemical Systems. J. Math. Chem..

[28] Noszticzius Z., McCormick W. D. (1988). Estimation of the Rate Constant of
the Ag^+^ + Br^−^ → AgBr Reaction.
The Possibility
of Non-Bromide-Controlled Oscillations in the Belousov-Zhabotinskii
Reaction. J. Phys. Chem. A.

[29] Noyes R. M., Field R. J., Försterling H.-D., Körös E., Ruoff P. (1989). Controversial Interpretations of
Silver­(1+) Perturbation of the Belousov-Zhabotinskii
Reaction. J. Phys. Chem. A.

[30] Ganapathisubramanian N., Noyes R. M. (1982). Chemical
Oscillations and Instabilities. Part 49. Additional
Complexities during Oxidation of Malonic Acid in the Belousov-Zhabotinskii
Oscillating Reaction. J. Phys. Chem. A.

[31] Gyorgyi L., Turányi T., Field R. J. (1990). Mechanistic Details of the Oscillatory
Belousov-Zhabotinskii Reaction. J. Phys. Chem.
A.

[32] Brusa M. A., Perissinotti L. J., Colussi A. (1985). Electron Spin Resonance Kinetic Studies
of Malonyl Radical Self-Decay and Oxidation Reactions by Cerium­(IV)
and Bromate in Acid Aqueous Media. The Role of Free Radicals in the
Belousov-Zhabotinskii Oscillator. J. Phys. Chem.
A.

[33] Noszticzius Z., Gáspár V., Försterling H.-D. (1985). Experimental Test for the Control
Intermediate in the Belousov-Zhabotinskii (BZ) Reaction. J. Am. Chem. Soc..

[34] Zhang J., Zhou L., Ouyang Q. (2007). Estimation of the Activation Energy
in the Belousov-Zhabotinsky Reaction by Temperature Effect on Excitable
Waves. J. Phys. Chem. A.

[35] Howell L., Osborne E., Franklin A., Hébrard E. (2021). Pattern Recognition
of Chemical Waves: Finding the Activation Energy of the Autocatalytic
Step in the Belousov-Zhabotinsky Reaction. J.
Phys. Chem. B.

[36] Muzika F., Schreiberová L., Schreiber I. (2020). Advanced Chemical
Computing Using
Discrete Turing Patterns in Arrays of Coupled Cells. Front. Chem..

[37] Yoshida R., Takahashi T., Yamaguchi T., Ichijo H. (1996). Self-Oscillating Gel. J. Am.
Chem. Soc..

[38] Ren L., Yuan L., Gao Q., Teng R., Wang J., Epstein I. R. (2020). Chemomechanical
Origin of Directed Locomotion Driven
by Internal Chemical Signals. Sci. Adv..

[39] ter
Harmsel M., Maguire O., Runikhina S., Wong A. S. Y., Huck W. T. S., Harutyunyan S. R. (2023). A Catalytically
Active Oscillator Made from Small Organic Molecules. Nature.

[40] Gyorgyi L., Field R. J. (1991). Simple Models of Deterministic Chaos
in the Belousov-Zhabotinskii
Reaction. J. Phys. Chem. A.

[41] Dourvas N. I., Sirakoulis G., A A. (2017). Cellular Automaton Belousov-Zhabotinsky
Model for Binary Full Adder. Int. J. Bifurcation
Chaos.

[42] Chen Y.-F., Lin H.-P., Sun S. S., Jwo J.-J. (1996). Kinetic Study of
the Ce­(III)- or Ferroin-Catalyzed Belousov-Zhabotinsky Reaction with
Ethyl- or Butyl-Malonic Acid. Int. J. Chem.
Kinet..

[43] Yu Y.-O., Jwo J.-J. (2000). Kinetic Study of
the Ce­(III)-, Mn­(II)-, or Ferroin-Catalyzed
Belousov-Zhabotinsky Reaction with Allylmalonic Acid. J. Chin. Chem. Soc..

[44] Chantler C. T., Bunker G., D’Angelo P., Diaz-Moreno S. (2024). X-ray Absorption
Spectroscopy. Nat. Rev. Methods Primers.

[45] Frateloreto F., Tavani F., Di Berto Mancini M., Del Giudice D., Capocasa G., Isabelle K., Kieffer I., Lanzalunga O., Lanzalunga O., Di Stefano S., Di Stefano S., D’Angelo P. (2022). Following a Silent Metal Ion: A Combined
X-ray Absorption
and Nuclear Magnetic Resonance Spectroscopic Study of the Zn^2+^ Cation Dissipative Translocation between Two Different Ligands. J. Phys. Chem. Lett..

[46] Tavani F., Capocasa G., Martini A., Sessa F., Di Stefano S., Lanzalunga O., D’Angelo P. (2021). Activation of C-H Bonds by a Nonheme
Iron­(iv)-Oxo Complex: Mechanistic Evidence through a Coupled EDXAS/UV-Vis
Multivariate Analysis. Phys. Chem. Chem. Phys..

[47] Tavani F., Frateloreto F., Del Giudice D., Capocasa G., Di Berto
Mancini M., Busato M., Lanzalunga O., Di Stefano S., D’Angelo P. (2024). Coupled X-ray Absorption/UV–vis
Monitoring of a Prototypical Oscillating Reaction. J. Phys. Chem. Lett..

[48] Capocasa G., Di Berto Mancini M., Frateloreto F., Del Giudice D., Lanzalunga O., Di Stefano S., D’Angelo P., Tavani F. (2025). A Combined X-ray Absorption and UV-Vis
Spectroscopic
Study of the Iron-Catalyzed Belousov-Zhabotinsky Reaction. J. Phys. Chem. Lett..

[49] Ravel B., Newville M. (2005). ATHENA, ARTEMIS, HEPHAESTUS: data
analysis for X-ray
absorption spectroscopy using IFEFFIT. J. Synchrotron
Rad..

[50] Del
Giudice D., Tavani F., Di Berto Mancini M., Frateloreto F., Busato M., Oliveira De Souza D., Cenesi F., Lanzalunga O., Di Stefano S., D’Angelo P. (2022). Two Faces of the Same Coin: Coupling X-Ray Absorption
and NMR Spectroscopies to Investigate the Exchange Reaction Between
Prototypical Cu Coordination Complexes. Chem.
- Eur. J..

[51] Timoshenko J., Haase F. T., Saddeler S., Rüscher M., Jeon H. S., Herzog A., Hejral U., Bergmann A., Schulz S., Roldan Cuenya B. (2023). Deciphering
the Structural and Chemical
Transformations of Oxide Catalysts during Oxygen Evolution Reaction
Using Quick X-ray Absorption Spectroscopy and Machine Learning. J. Am. Chem. Soc..

[52] Timoshenko J., Anspoks A., Cintins A., Kuzmin A., Purans J., Frenkel A. I. (2018). Neural Network Approach for Characterizing Structural
Transformations by X-Ray Absorption Fine Structure Spectroscopy. Phys. Rev. Lett..

[53] Migliorati V., Mancini G., Tatoli S., Zitolo A., Filipponi A., De Panfilis S., Di Cicco A., D’Angelo P. (2013). Hydration
Properties of the Zn^2+^ Ion in Water at High Pressure. Inorg. Chem..

[54] D’Angelo P., Di Nola A., Mangoni M., Pavel N. V. (1996). An Extended X-ray
Absorption Fine Structure Study by Employing Molecular Dynamics Simulations:
Bromide ion in Methanolic Solution. J. Chem.
Phys..

[55] D’Angelo P., Migliorati V., Guidoni L. (2010). Hydration Properties of the Bromide
Aqua Ion: the Interplay of First Principle and Classical Molecular
Dynamics, and X-ray Absorption Spectroscopy. Inorg. Chem..

[56] D’Angelo P., Di Cicco A., Filipponi A., Pavel N. V. (1993). Double-Electron
Excitation Channels at the Br K Edge of HBr and Br_2_. Phys. Rev. A.

[57] Burattini E., D’Angelo P., Di Cicco A., Filipponi A., Pavel N. V. (1993). Multiple scattering X-ray absorption analysis of simple
brominated hydrocarbon molecules. J. Phys. Chem.
A.

[58] D’Angelo P., Di Nola A., Filipponi A., Pavel N. V., Roccatano D. (1994). An Extended
X-ray Absorption Fine Structure Study of Aqueous Solutions by Employing
Molecular Dynamics Simulations. J. Chem. Phys..

[59] Ferlat G., Miguel A. S., Jal J. F., Soetens J. C., Bopp P. A., Daniel I., Guillot S., Hazemann J. L., Argoud R. (2001). Hydration
of the Bromine Ion in a Supercritical 1:1 Aqueous Electrolyte. Phys. Rev. B.

[60] Neese F. (2012). The ORCA program
system. WIREs Rev. Comput. Mol. Sci..

[61] Joly Y. (2001). X-ray Absorption
Near-Edge Structure Calculations Beyond the Muffin-Tin Approximation. Phys. Rev. B.

[62] Bunău O., Joly Y. (2009). Self-consistent Aspects of X-ray
Absorption Calculations. J. Phys: Condes. Matter.

[63] Parrilla-Gutierrez J.
M., Sharma A., Tsuda S., Cooper G. J. T., Aragon-Camarasa G., Donkers K., Cronin L. (2020). A Programmable Chemical Computer
with Memory and Pattern Recognition. Nat. Commun..

[64] Synchronization in Lattices of Coupled Oscillators. Phys. D: Nonlinear Phenomena 1997, 103, 442−451 10.1016/S0167-2789(96)00276-X.

