# Supplementary material for: Concentration-Dependent Evolution of the Belousov–Zhabotinsky Reaction as Determined by X‑ray Absorption and UV–Vis Spectroscopies
Source: J Phys Chem B. 2025 Oct 3;129(41):10838–45. doi: 10.1021/acs.jpcb.5c04472 (PMC12536401; doi:10.1021/acs.jpcb.5c04472)
Supplement: Supplementary file 1 [file jp5c04472_si_001.pdf]

Supporting Information: Concentration-dependent evolution of the Belousov-Zhabotinsky reaction as determined by X-ray absorption and UV-Vis spectroscopies

Francesco Tavani,* Giorgio Capocasa, Marika Di Berto Mancini, Federico Frateloreto, Daniele Del Giudice, Osvaldo Lanzalunga, Stefano Di Stefano, and Paola D'Angelo*

Dipartimento di Chimica, Università degli Studi di Roma "La Sapienza", P.le A. Moro 5, I-00185 Rome, Italy

E-mail: francesco.tavani@uniroma1.it; p.dangelo@uniroma1.it

Contents

1 Materials **S-3**

1.1 BZ reaction conditions S-3

2 Methods **S-3**

2.1 X-ray Absorption measurements S-3

2.2 Decomposition of the Br K-edge XANES data S-4

2.3 Determination of the number of principal components through the scree plot statistical test S-6

2.4 FKN model S-6

2.5 Theoretical method S-6

3 Supplementary Figures S1–S4 **S-8**

References **S-12**

1 Materials

1.1 BZ reaction conditions

The Ce 17 mM BZ reaction was investigated by independent UV-Vis and Br K-edge XAS measurements. The Ce 17 mM BZ reactions were carried out by mixing $Ce(NH_4)_4(SO_4)_4$ (17.0 mM), H_2SO_4 (0.48 M), $NaBrO_3$ (80 mM), KBr (8.0 mM) and malonic acid (MA, 50 mM) in aqueous solution at 25 °C.

The BZ reaction involving Ce 34 mM was carried by mixing $Ce(NH_4)_4(SO_4)_4$ (34.0 mM), H_2SO_4 (1.7 M), $NaBrO_3$ (70.2 mM), KBr (5.6 mM) and MA (70.0 mM) in aqueous solution at 25 °C (for additional details, we refer the reader to Ref.[S1]).

The BZ reaction involving Fe 3 mM was carried out by mixing ferroine (3.0 mM), H_2SO_4 (0.48 M), $NaBrO_3$ (80 mM), KBr (8.0 mM) and allylmalonic acid (50 mM) in aqueous solution at 25 °C (for additional details, we refer the reader to Ref.[S2]).

All BZ reactions described in this work were performed in initially homogeneized, unstirred solutions, obtained by sequentially adding the precursor solutions into the reaction cell. The reaction cell used to perform the UV-Vis independent measurements (for all described BZ reactions) is shown in Figure S1 and had height, inner width and inner depth dimensions of 52 mm, 9.5 mm and 1 mm, respectively. The cell employed to monitor the BZ reaction with the XAS independent measurements was obtained by sealing kapton windows on an Aluminum alloy cell possessing the same dimensions listed above.

2 Methods

2.1 X-ray Absorption measurements

The Br K-edge XAS spectra shown in this work were collected at room temperature in transmission mode at the Elettra Synchrotron (Trieste, Italy) on the XAFS beamline. The storage ring was operating at 2 GeV with an optimal storage beam current between 300 and

130 mA. The Aluminum alloy cell was sealed using Kapton film windows of 0.5 mm. The time-resolved XANES spectra were collected by employing a fast scan mode available at the beamline.

All measured XAS spectra were directly normalized by means of the Athena software package,[S3] without applying additional spectral smoothing procedures. The manuscript plots were generated by means of custom python scripts.

2.2 Decomposition of the Br K-edge XANES data

The time-resolved XANES measurements yielded a series of spectra that were placed in a dataset matrix $\boldsymbol{D}$, where each column of $\boldsymbol{D}$ was identified with a spectrum measured at a given time t. Following Lambert-Beer's law, we decomposed the experimental data into the spectra associated to the key species and in their relative concentration profiles. To this end we employed the PyFitit code,[S4] a software that uses to such end an algorithm belonging to the MCR family.

The Singular Value Decomposition (SVD) equation provided the starting point for our analysis:

$$\boldsymbol{D} = \boldsymbol{U} \cdot \boldsymbol{\Sigma} \cdot \boldsymbol{V} + \boldsymbol{E} \tag{1}$$

where the product $\boldsymbol{U} \cdot \boldsymbol{\Sigma}$ contains a set of values that may be associated to the normalized absorption coefficients on its N columns, $\boldsymbol{\Sigma}$ is a diagonal matrix whose elements are sorted in decreasing order, while $\boldsymbol{V}$ can be interpreted as the concentration matrix. Lastly, the error matrix $\boldsymbol{E}$ represents the lack of fit between the experimental data matrix $\boldsymbol{D}$ and the reconstructed matrix $\boldsymbol{\mu} = \boldsymbol{U} \cdot \boldsymbol{\Sigma} \cdot \boldsymbol{V}$. The SVD deconvolution depends on the correct estimation of the number of components N present in the experimental spectral matrix. To this end, we evaluated the percentage error committed in reproducing the experimental data with an increasing number N of components (the results are shown in Figure 3b of the main

text). The percentage error function has been calculated with the following expression:

$$R(n) = \frac{\sum_{i=1}^{K}\sum_{j=1}^{m} |d_{ij} - \mu_{ij}^{PC=n}|}{\sum_{i=1}^{K}\sum_{j=1}^{m} |d_{ij}|} \times 100 \tag{2}$$

where d_{ij} and $\mu_{ij}^{PC=n}$ are the normalized absorbance values for the dataset and for the dataset reconstructed with N = n, respectively (K and m represent the number of acquired spectra and of the energy points, respectively, while $n = 1, 2, ...K$).

Once N is established, the approach implemented by PyFitIt requires the introduction of a transformation N × N matrix $\boldsymbol{T}$ in Equation 1, using the relation $\boldsymbol{I} = \boldsymbol{T} \cdot \boldsymbol{T}^{-1}$:

$$\boldsymbol{D} = \boldsymbol{U} \cdot \boldsymbol{\Sigma} \cdot \boldsymbol{T} \cdot \boldsymbol{T}^{-1} \cdot \boldsymbol{V} + \boldsymbol{E} \tag{3}$$

where the spectra belonging to the key species are given by $\boldsymbol{S} = \boldsymbol{U} \cdot \boldsymbol{\Sigma} \cdot \boldsymbol{T}$ and their concentration profiles by $\boldsymbol{C} = \boldsymbol{T}^{-1} \cdot \boldsymbol{V}$. Subsequently, the matrix elements T_{ij} of matrix $\boldsymbol{T}$ are modified by sliders to achieve $\boldsymbol{S}$ and $\boldsymbol{C}$ which have chemical and physical meaning. One can then write:

$$\boldsymbol{D} = \boldsymbol{S} \cdot \boldsymbol{C} + \boldsymbol{E} \tag{4}$$

The unknown number of T_{ij} elements of $\boldsymbol{T}$ is in principle equal to N^2. In order to reduce such ambiguity, the XANES spectrum of the BrO_3^- and Br^- references were constrained to coincide with two of the three extracted components. This operation allows the reduction of the number of unknown T_{ij} elements from N^2 to $N^2 - N$. In our investigation, a 3 × 3 matrix $\boldsymbol{T}$ containing 9 elements was employed to retrieve the spectral and concentration profiles of the three key species contributing to the XANES spectra measured during the BZ reaction.

2.3 Determination of the number of principal components through the scree plot statistical test

We applied a scree plot test on the XANES Br K-edge data to assess the number of pure species present in the reaction mixture.

One may show that:

$$\lambda_i = \frac{\sigma_{ii}^2}{m-1} \tag{5}$$

where σ_{ii} are the singular values extracted by the SVD procedure and λ_i are the eigenvalues of the covariance matrix of $\mathbf{D}$ (evaluated for m energy steps) relative to every i-th component. These values correspond to the variance of each principal component (PC).[S4,S5] Consequently, the components with a high σ_{ii} value contribute significantly to the dataset reconstruction, while those with a small σ_{ii} value are associated to noise.

In the scree plot, the singular values relative to each principal component are plotted against the number of PCs. The presence of an elbow in such curve separates the signal and noise related components.

2.4 FKN model

The FKN model of the iron-catalyzed Belousov-Zhabotinsky reaction is outlined in Table S1 below.[S6]

2.5 Theoretical method

Theoretical calculations were performed employing the ORCA code.[S7] The geometry of BrMA was optimized at the Density Functional Theory (DFT) theory level employing the B3LYP functional, the D3BJ dispersion correction, with a ZORA-def2-TZVP basis set. The FDMNES code has been employed to perform the XANES theoretical data analysis using the muffin-tin approximation for the potential and including quadrupole transitions in the

Table S1: Key processes involved in the FKN mechanism of the iron ion catalyzed-Belousov-Zhabotinsky reaction (MA = lmalonic acid, BrMA = bromomalonic acid).[S6]

Step	Reaction
R1	$HOBr + H^+ + Br^- \rightleftharpoons Br_2 + H_2O$
R2	$HBrO_2 + H^+ + Br^- \rightleftharpoons 2HOBr$
R3	$BrO_3^- + 2H^+ + Br^- \rightleftharpoons HBrO_2 + HOBr$
R4	$2HBrO_2 \rightleftharpoons BrO_3^- + HOBr + H^+$
R4b	$HBrO_2 + H_2BrO_2^+ \rightarrow BrO_3^- + HOBr + 2H^+$
R5a	$BrO_3^- + HBrO_2 + H^+ \rightleftharpoons Br_2O_4 + H_2O$
R5b	$Br_2O_4 \rightleftharpoons 2BrO_2^\bullet$
R6	$BrO_2^\bullet + Fe(phen)_3^{2+} + H^+ \rightleftharpoons HBrO_2 + Fe(phen)_3^{3+}$
R8a	$Br_2 + MA(enol) \rightarrow BrMA + Br^- + H^+$
R8b	$HOBr + MA(enol) \rightarrow BrMA + H_2O$
R9	$Fe(phen)_3^{3+} + BrMA \rightleftharpoons P1 + Fe(phen)_3^{2+} + Br^- + 2H^+$
R10	$Fe(phen)_3^{3+} + MA \rightarrow P2 + H^+ + Fe(phen)_3^{2+}$
E	$MA \rightleftharpoons MA(enol)$
A1	$H_2BrO_2^+ \rightleftharpoons HBrO_2 + H^+$

calculations.[S8,S9] FDMNES represents originally a one-electron approach and is based on the ab-initio calculations of the electronic structure and on the resolution of the discretized radial Schrodinger equation. The FDM approach is attractive for the simulation of the photoelectron wave function beyond 100eV above the absorption edge.

3 Supplementary Figures S1–S4

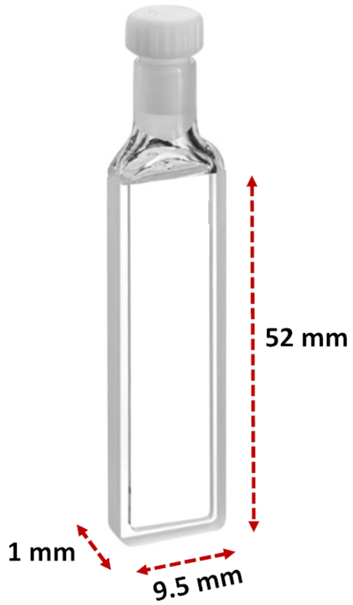

Figure S1: Cell employed to follow the Ce 17 mM BZ reaction by means of UV-Vis spectroscopy.[S1,S2] An Aluminum alloy cell with same dimensions as those listed in the figure and Kapton windows was employed to monitor the BZ reaction through XANES measurements.

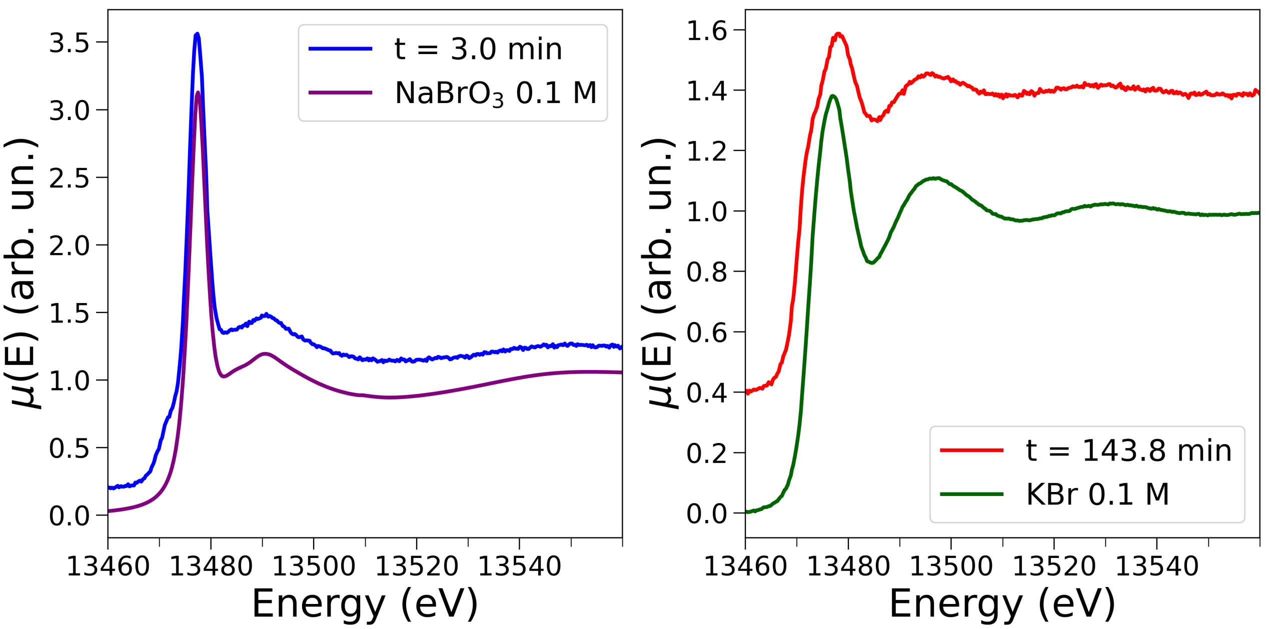

Figure S2: Br K-edge XAS spectra of the investigated BZ reaction measured at selected times from reaction start compared to the XAS spectra of $NaBrO_3$ (left panel) and KBr 0.1 M (right panel) aqueous solutions.

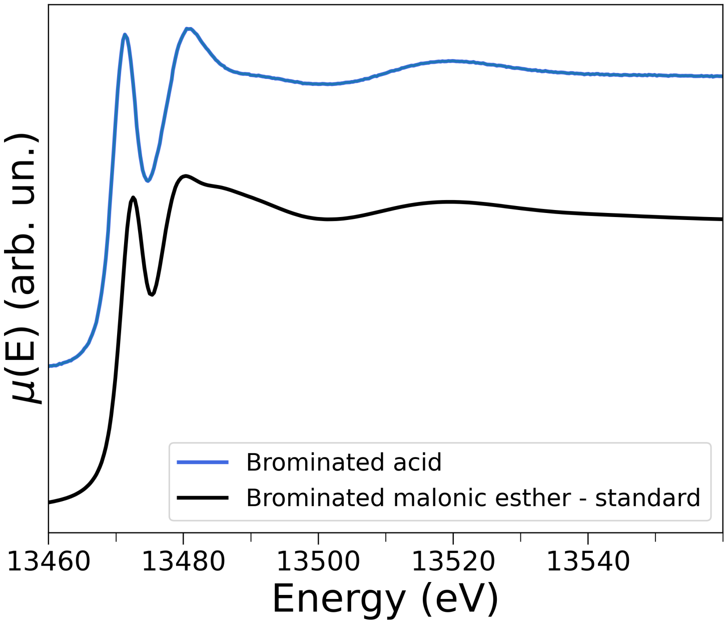

Figure S3: Br K-edge XAS spectrum of the reaction intermediate assigned to the BrMA species (light blue line) compared to that of a diethyl bromomalonate 0.1 M methanol solution (black line).

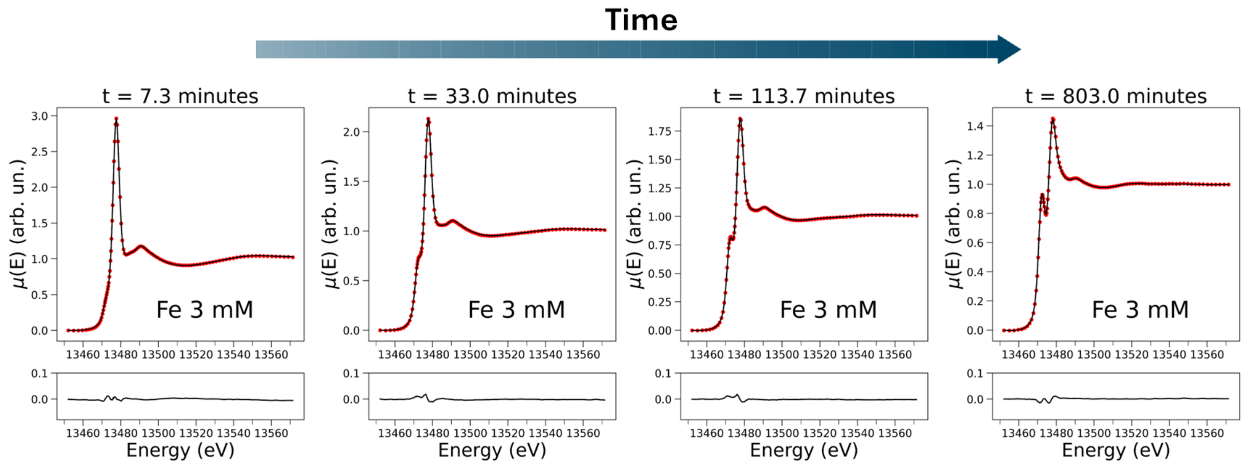

Figure S4: Statistical reconstruction of XANES spectra recorded while carrying out the BZ reaction in the presence of Fe 3 mM.[S2] Selected experimental XANES spectra (dotted red) are displayed at given times from reaction start along with their corresponding reconstructed XANES spectra obtained by employing N=3 PCs (solid black). The absolute errors between the experimental and reconstructed curves are reported below each XANES spectrum.

References

(S1) Tavani, F.; Frateloreto, F.; Del Giudice, D.; Capocasa, G.; Di Berto Mancini, M.; Busato, M.; Lanzalunga, O.; Di Stefano, S.; D'Angelo, P. Coupled X-ray Absorption/UV-vis Monitoring of a Prototypical Oscillating Reaction. *J. Phys. Chem. Lett.* **2024**, *15*, 7312–7319.

(S2) Capocasa, G.; Di Berto Mancini, M.; Frateloreto, F.; Del Giudice, D.; Lanzalunga, O.; Di Stefano, S.; D'Angelo, P.; Tavani, F. A Combined X-ray Absorption and UV-Vis Spectroscopic Study of the Iron-Catalyzed Belousov-Zhabotinsky Reaction. *J. Phys. Chem. Lett.* **2025**, *16*, 1840–1846.

(S3) Ravel, B.; Newville, M. *ATHENA*, *ARTEMIS*, *HEPHAESTUS*: data analysis for X-ray absorption spectroscopy using *IFEFFIT*. *J. Synchrotron Rad.* **2005**, *12*, 537–541.

(S4) Martini, A.; Guda, S.; Guda, A.; Smolentsev, G.; Algasov, A.; Usoltsev, O.; Soldatov, M.; Bugaev, A.; Rusalev, Y.; Lamberti, C.; Soldatov, A. PyFitit: The Software for Quantitative Analysis of XANES Spectra Using Machine-Learning Algorithms. *Comput. Phys. Comm.* **2019**, 107064.

(S5) Markovsky, I. Structured low-rank approximation and its applications. *Automatica* **2008**, *44*, 891–909.

(S6) Zars, E.; Glaser, R.; Downing, M.; Chicone, C. Measurements and Simulations of the Acidity Dependence of the Kinetics of the Iron-Catalyzed Belousov-Zhabotinsky Reaction: Proton-Catalysis in the Electron Transfer Reaction Involving the $[Fe(phen)_3]^{3+}$ Species. *J. Phys. Chem. A* **2018**, *122*, 6183–6195.

(S7) Neese, F. The ORCA program system. *Wiley Interdiscip. Rev. Comput. Mol. Sci.* **2012**, *2*, 73–78.

(S8) Joly, Y. X-ray Absorption Near-Edge Structure Calculations Beyond the Muffin-Tin Approximation. *Phys Rev B* **2001**, *63*, 125120.

(S9) Bunău, O.; Joly, Y. Self-consistent Aspects of X-ray Absorption Calculations. *J. Phys: Condes. Matter* **2009**, *21*, 345501.
